# Using Recombinant Lactococci as an Approach to Dissect the Immunomodulating Capacity of Surface Piliation in Probiotic *Lactobacillus rhamnosus* GG

**DOI:** 10.1371/journal.pone.0064416

**Published:** 2013-05-14

**Authors:** Ingemar von Ossowski, Taija E. Pietilä, Johanna Rintahaka, Elina Nummenmaa, Veli-Matti Mäkinen, Justus Reunanen, Reetta Satokari, Willem M. de Vos, Ilkka Palva, Airi Palva

**Affiliations:** 1 Department of Veterinary Biosciences, Faculty of Veterinary Medicine, University of Helsinki, Helsinki, Finland; 2 Functional Foods Forum, University of Turku, Turku, Finland; 3 Laboratory of Microbiology, Wageningen University, Wageningen, The Netherlands; 4 Department of Bacteriology and Immunology, Haartman Institute, University of Helsinki, Helsinki, Finland; Institut Pasteur de Lille, France

## Abstract

Primarily arising from their well understood beneficial health effects, many lactobacilli strains are considered good candidates for use as probiotics in humans and animals. Lactobacillar probiosis can itself be best typified by the *Lactobacillus rhamnosus* GG strain, which, with its well-documented clinical benefits, has emerged as one of the most widely used probiotics in the food and health-supplement industries. Even so, many facets of its molecular mechanisms and limitations as a beneficial commensal bacterium still remain to be thoroughly explored and dissected. Because *L. rhamnosus* GG is one of only a few such strains exhibiting surface piliation (called SpaCBA), we sought to examine whether this particular type of cell-surface appendage has a discernible immunomodulating capacity and is able to trigger targeted responses in human immune-related cells. Thus, presented herein for this study, we recombinantly engineered *Lactococcus lactis* to produce native (and pilin-deleted) SpaCBA pili that were assembled in a structurally authentic form and anchored to the cell surface, and which had retained mucus-binding functionality. By using these recombinant lactococcal constructs, we were able to demonstrate that the SpaCBA pilus can be a contributory factor in the activation of Toll-like receptor 2-dependent signaling in HEK cells as well as in the modulation of pro- and anti-inflammatory cytokine (TNF-α, IL-6, IL-10, and IL-12) production in human monocyte-derived dendritic cells. From these data, we suggest that the recombinant-expressed and surface-anchored SpaCBA pilus, given its projected functioning in the gut environment, might be viewed as a new microbe-associated molecular pattern (MAMP)-like modulator of innate immunity. Accordingly, our study has brought some new insight to the molecular immunogenicity of the SpaCBA pilus, thus opening the way to a better understanding of its possible role in the multifaceted nature of *L. rhamnosus* GG probiosis within the human gut.

## Introduction

Gram-positive lactobacilli are recurrent members of the gut commensal community and among the earliest inhabitants of the gastrointestinal (GI) tract [1]. Stemming from the century-old Metchnikoff concept of lactobacilli, a certain proportion of intestinal strains, as well as isolates from other habitats, are used routinely now as probiotics in humans and animals. Characteristically, such bacteria are understood to possess a strong propensity to help promote good health as well as alleviate a variety of health problems [2]–[4]. However, to varying extents, these types of lactobacilli are associated mainly with the fecal stream and exist only briefly in the gut [5]–[7]. Thus, many lactobacilli classified as probiotic are transient inhabitants and so part of a temporal gut microbiome, as they find it difficult to permanently colonize a specific niche in the intestine. However, despite some genetic shortcomings associated with their overall adhesion capacity, when such lactobacilli are replenished constantly in high numbers in the gut, they can perform key functions in sustaining a normal microbial balance and in helping to preclude certain pathogen-borne infections [8].

Perhaps most significantly, many recent studies have begun to scrutinize how important probiotic lactobacilli are as transitory, but also targeted, inducers of protective host immunity, which in many instances involve the various defense mechanisms of the innate and adaptive immune systems [9]. For example, as it has been reviewed in the literature recently [10]–[12], bacterial cell-induced immunomodulation involving lactobacilli includes immediate innate responses that can inhibit inflammation, regulate Toll-like receptor (TLR) signaling, trigger natural killer (NK) cell release, and stimulate maturation of antigen-presenting dendritic cells (DCs). Delayed adaptive immune responses that are induced by lactobacilli have typically involved activating lymphocyte proliferation, releasing gut-specific immunoglobulin, and affecting the T helper-1 and -2 (Th1/Th2) balance [10]–[12]. Up to now, it is commonly accepted these immune functions are induced by probiotic lactobacilli via mechanisms that are likely strain-specific and multifactorial in nature. Notwithstanding, many studies are beginning to credit a variety of proteinaceous and carbohydrate-like cell-surface components as being contributory to the specific manner by which certain of these lactobacillar strains can elicit host immune-related responses (for review, see [13]).

*Lactobacillus rhamnosus* GG is perhaps one of the best-known paradigms of lactobacillar probiosis, having over the many years been thoroughly investigated, both *in vitro* and *in vivo* (for review, see [14]). Originally recovered from a healthy human gut, *L. rhamnosus* GG has gone on to be widely used in various foods and dietary supplements for its perceived health benefitting properties. Consequently, it is recognized as one of the stalwart strains of the probiotic industry. However, in spite of this, the molecular mechanisms and limitations of *L. rhamnosus* GG as a beneficial commensal bacterium are still far from completely understood. For instance, while the various clinical benefits of *L. rhamnosus* GG have already become well documented in the literature, relatively few studies have characterized those specific traits responsible for its immunomodulating capacity. Nonetheless, some recent work has started to link the different immune-related actions to a particular set of interesting niche factors. As an example, two soluble proteins released from *L. rhamnosus* GG cells, called p75 and p40 (also known as Msp1 and Msp2, respectively [15], [16]) have been shown to be responsible for the previously established ability of cells to deter cytokine-stimulated cell death in the intestinal epithelium, and which as a protective mechanism involves the activation of the anti-apoptotic PI3K/Akt signaling pathway [17]–[20]. Another interesting example involves the galactose-rich exopolysaccharides (EPS) surrounding *L. rhamnosus* GG cells, in which it was reported that because sub-inhibitory levels of the antimicrobial peptide LL-37 could trigger EPS production in cells, this layer of carbohydrate molecules is thought to have a protective shielding function toward host innate immune effectors [21]. Finally, one more example involves the lipoteichoic acids (LTAs) in the cell wall of *L. rhamnosus* GG, which were recently shown to stimulate TLR2/6-dependent activation of NF-κB (nuclear factor-kappa beta) signaling in an human embryonic kidney cell line (HEK293T) as well as induce pro-inflammatory cytokine interleukin-8 (IL-8) mRNA expression in human intestinal epithelial Caco-2 cells [22].

Somewhat recently, we identified *L. rhamnosus* GG as one of the first probiotic strains known to be piliated [23]. This finding in itself was viewed as intriguing given that previously the occurrence of pili amongst Gram-positive bacteria was found primarily in pathogenic strains and normally associated with their virulence (for review, see [24]). We further revealed that the overall structural arrangement of the *L. rhamnosus* GG pilus (called SpaCBA) bears close resemblance to the typical three-pilin subunit architecture of those already determined from other piliated Gram-positive bacteria [25]. Most importantly, functionally attributed to the SpaCBA pilus were the abilities to adhere strongly to both human-derived intestinal mucus [23], [26] and Caco-2 cells [27] as well as to promote biofilm growth [27], all of which might be considered the needed phenotypic traits of probiotic lactobacilli that can likely help support increased adhesion and longevity in the gut. However, although a functional correlation between cell mucoadhesiveness and pili in *L. rhamnosus* GG was basically established, a recent study using knockout mutants had demonstrated that the SpaCBA pilus has also an immunomodulating function in intestinal epithelial cells, albeit an indirect one [27]. Therein, it was found that the SpaCBA pilus has an apparent dampening effect on the mRNA levels of cytokine IL-8, but whose direct induction was triggered initially by LTA, possibly being mediated through a TLR2-dependent mechanism. Still, a more recent study has inferred the possibility that SpaCBA piliation might have a role in the TLR2-mediated NF-κB signaling that was induced by *L. rhamnosus* GG, but not by two nonpiliated *Lactobacillus casei* strains [28].

Because the potential role of pili from *L. rhamnosus* GG in triggering key signaling pathways of inflammation is less than resolved and even much less understood at the present time, we sought to investigate further the immunomodulatory functioning of the SpaCBA pilus by assessing its ability to stimulate certain innate immune responses in human immune-related cells. For this, instead of utilizing the traditional knockout-mutant approach, we opted for a different strategy and genetically engineered the food-grade *Lactococcus lactis* NZ9000 strain to produce SpaCBA pili. *Lactococcus* species are themselves well known to be the cloning workhorses of lactic acid bacteria (LAB) and have been employed frequently in studies as the host for the recombinant expression of many different proteins (for review, see [29]–[31]), including pili from Gram-positive pathogens [32]–[34].

In our study presented herein, the cloning of *L. lactis* NZ9000 containing the *L. rhamnosus* GG *spaCBA* pilus operon was achieved to produce the recombinant SpaCBA pilus in its native (and pilin-deleted) functional form that was both authentic structurally and fully assembled with all pilin subunits. Accordingly, by using these recombinant constructs of piliated lactococci, the putative capacity of SpaCBA pili for activating the TLR2-dependent NF-κB signaling in HEK cells and modulating human monocyte-derived dendritic cell (moDC) cytokine production was explored. From these results, we suggest that the role of the surface-anchored SpaCBA pilus can be expanded beyond just a simple mucus-adherent function to one that now includes prospective involvement as an adhesive potentiating modulator of innate immunity, and which one can speculate might conceivably be manifested in the gut environment.

## Results and Discussion

### Lactococcal surface expression of wild-type and pilin-deleted SpaCBA pili

**Figure 1:**
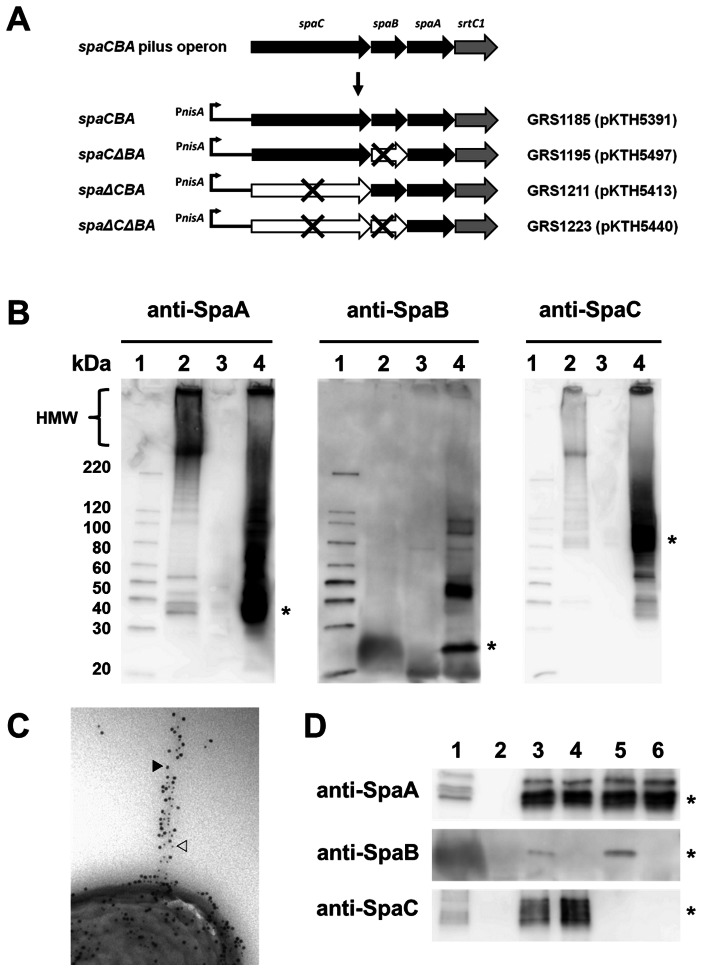
Characterization of recombinant-expressed *L. rhamnosus* GG SpaCBA pili in *L. lactis*. (A) The coding region of the *L. rhamnosus* GG *spaCBA* pilus operon and a summary of the nisin promoter-regulated (P*nisA*) WT and pilin-deleted SpaBCA-piliated recombinant constructs in *L. lactis* are depicted schematically. Genes for the pilin subunits (*spaA*, *spaB*, and *spaC*) and pilin-specific sortase (*srtC1*), including those genes that are deleted (×) in the recombinant constructs, are indicated. Identities for the various SpaCBA-piliated lactococcal constructs (GRS1185, GRS1195, GRS1211, and GRS1223), including the *spaCBA* pilus operon-containing plasmids they each propagate (pKTH5391, pKTH5497, pKTH5413, and pKTH5440, respectively), are indicated. (B) Immunoblot analysis of cell wall-extracted proteins recovered from *L. rhamnosus* GG (lane 2), the empty vector (pKTH5080)-carrying GRS1052 *L. lactis* construct (lane 3), and the nisin-induced WT SpaCBA-piliated GRS1085 *L. lactis* construct (lane 4) probed with the various pilin-specific antisera (anti-SpaA, anti-SpaB, or anti-SpaC, as indicated). Asterisks on the right of each immunoblot indicate the presumed location of monomeric SpaA, SpaB, and SpaC pilin proteins. The compressed and laddered region of high-molecular-weight (HMW) protein bands representing various lengths of pili is indicated on the left. Molecular weight markers (lane 1) and their positions are indicated on the left. (C) Immunogold double-labeling electron microscopy of WT SpaCBA-piliated lactococcal cells (GRS1185) using SpaA antiserum with protein A-10-nm gold particles as well as SpaC antiserum with protein A-5-nm gold particles is shown. The location of 5-nm (white arrow) and 10-nm (black arrow) gold particles on the pilus structure are indicated. (D) Immunoblot analysis of cell wall-extracted proteins recovered from *L. rhamnosus* GG (lane 1) and the empty vector (pKTH5080)-carrying GRS1052 *L. lactis* construct (lane 2), as well as the nisin-induced WT (GRS1085) (lane 3), SpaB-deleted (GRS1195) (lane 4), SpaC-deleted (GRS1211) (lane 5), and SpaB- and SpaC-deleted (GRS1223) (lane 6) piliated *L. lactis* constructs probed with anti-SpaA, anti-SpaB, or anti-SpaC sera, each of which is indicated on the left of the respective immunoblot. Positions of monomeric SpaA (∼31 kDa), SpaB (∼21 kDa), and SpaC (∼91 kDa) protein bands are marked by an asterisk on the right of corresponding immunoblots.

Genes for the SpaCBA pilus are arranged in the *L. rhamnosus* GG genome as an operon (Figure 1A) and encode for three pilin proteins (i.e., the major SpaA and the minor SpaB and SpaC subunits) and the pilin-specific sortase, an enzyme that is needed for pilus polymerization [23]. Based on our previous electron microscopy-derived interpretations, the structural makeup of the fully assembled SpaCBA pilus consists of a polymerized backbone built of SpaA subunits, SpaC at the pilus tip, and the basal SpaB, of which the latter two ancillary subunits are also positioned irregularly along the length of the pilus structure [23], [25].

To facilitate our studies on the immunomodulatory capacity of SpaCBA pili, we first engineered a plasmid construct that allows expression in *L. lactis* NZ9000 of a recombinant form of the wild-type (WT) SpaCBA pilus similar in composition to that described above. Here, the coding region of the *spaCBA* pilus operon was inserted into a nisin-inducible expression vector (pKTH5080) to generate recombinant plasmid pKTH5391, which was propagated as the GRS1185 lactococcal construct (Figure 1A). To establish whether pilus production took place after this lactococcal construct was induced with nisin, cell wall-extracted proteins were recovered, analyzed by immunoblotting with pilin-specific antiserum (anti-SpaA, anti-SpaB, or anti-SpaC), and then examined for a characteristic high-molecular-weight (HMW) pattern, in which compressed and laddered protein bands correspond to different lengths of pili.

As shown in Figure 1B, HMW protein was observed on immunoblots of cell wall-extracted proteins from nisin-induced cells of GRS1185 with all individual anti-pilin sera (lane 4). The most intense blotting signal was detected when anti-SpaA and anti-SpaC sera were each used to probe the blots and rather unlike that of SpaB-specific antibody, which in comparison gave a slightly weaker response. As a positive control (lane 2), the same sort of HMW banding pattern was also observed for cell wall protein extracts from *L. rhamnosus* GG cells, although, consistent with our earlier findings for SpaCBA pili [25], no HMW bands were visible when anti-SpaB serum was used. However, with all anti-pilin sera used, bands corresponding to the molecular weight size of the monomeric form of the SpaA (∼30 kDa), SpaB (∼20 kDa), and SpaC (∼90 kDa) pilins were detected in GRS1185 cell wall protein extracts (Figure 1B, lane 4) and as had been found in our previous work [25]. Expectedly, no such bands for HMW or monomeric protein could be detected in the negative control empty vector-carrying GRS1052 construct (lane 3). Altogether, these immunoblot data indicate that each of the three pilin subunits (SpaA, SpaB, and SpaC) is a structural component of an expressed and fully assembled cell wall-anchored WT pilus in *L. lactis*.

To provide convincing visual evidence of surface localization for recombinant WT SpaCBA pili in nisin-induced lactococcal cells, we used immunogold labeling of pilin subunits and transmission electron microscopy. Here, for example, the GRS1185 construct is seen to possess a pilus structure extending outwardly from the cell surface, which, based on detection with SpaA and SpaC antisera in a double-labeling experiment (Figure 1C) and as would be expected [25], is composed of a multitude of pilin subunits. On the other hand, the use of anti-SpaB serum to visualize recombinant pili proved less achievable given that rather few protein A-gold particles were observed to be associated within each pilus fiber (data not shown). Nonetheless, this was in line with not only our immunoblotting results (see above), but also our previous work on *L. rhamnosus* GG [25], in which we found that SpaB is more underrepresented as a structural constituent within the pilus backbone than the other two pilin types and instead mainly buried within the cell wall as a basal subunit.

We also constructed three piliated deletion mutants in lactococci, these of which include the SpaB-deleted GRS1195, SpaC-deleted GRS1211, and double SpaB/SpaC-deleted GRS1223 constructs (Figure 1A). In order to corroborate the absence of expression for deleted pilins in these mutant constructs during nisin-induced conditions, each was analyzed for their respective monomeric proteins by immunblotting with all three pilin-specific antisera. As can be seen for GRS1195 cell wall protein extracts (Figure 1D, lane 4), aside from the predictable bands that were detected with anti-SpaA and anti-SpaC sera, a band was not observed when the blot was treated with anti-SpaB serum, indicating that the SpaB pilin was not being expressed in this lactoccocal construct. Likewise, analogous results were also obtained when blots of cell wall proteins from cells of the remaining two mutant constructs were probed with anti-pilin sera, which confirmed that SpaC in GRS1211 (Figure 1D, lane 5) and both SpaB and SpaC in GRS1223 (Figure 1D, lane 6) were not being produced in these cells. Results obtained for pilin antiserum blots of cell wall protein extracts from *L. rhamnosus* GG (Figure 1D, lane 1) and GRS1052 (Figure 1D, lane 2), which had been used as positive and negative controls, respectively, were as to be expected. Immuno-electron microscopic analyses for some of the pilin deletion mutants with SpaA antiserum indicated that these constructs were still able to display polymerized cell surface-localized pili (data not shown). However, given that the basal SpaB pilin is likely seen as the controlling factor that terminates the polymerization process and signals the covalent cell wall-attachment of pili [25], it is not certain whether this missing subunit in GRS1195 and GRS1223 causes pili in such cells to be only membrane bound through an interaction with the pilin-specific sortase.

### Mucus adhesion capacity of recombinant SpaCBA-piliated lactococcal constructs

Previously, we demonstrated that the adhesive interaction between *L. rhamnosus* GG and human intestinal mucus was, in addition to other surface adhesins [35], [36], strongly dependent upon the SpaCBA pilus, with the main mucus-binding determinant shown to be the SpaC pilin [23], [26]. Interestingly, an adherent but perhaps novel ability to bind mucus was also attributed to recombinant-produced basal SpaB protein, which unexpectedly involved electrostatic interactions [26]. With this in mind, we sought to authenticate lactococcal-expressed SpaCBA pili by assessing the level of mucoadhesiveness for the recombinant WT and various pilin deletion constructs.

**Figure 2:**
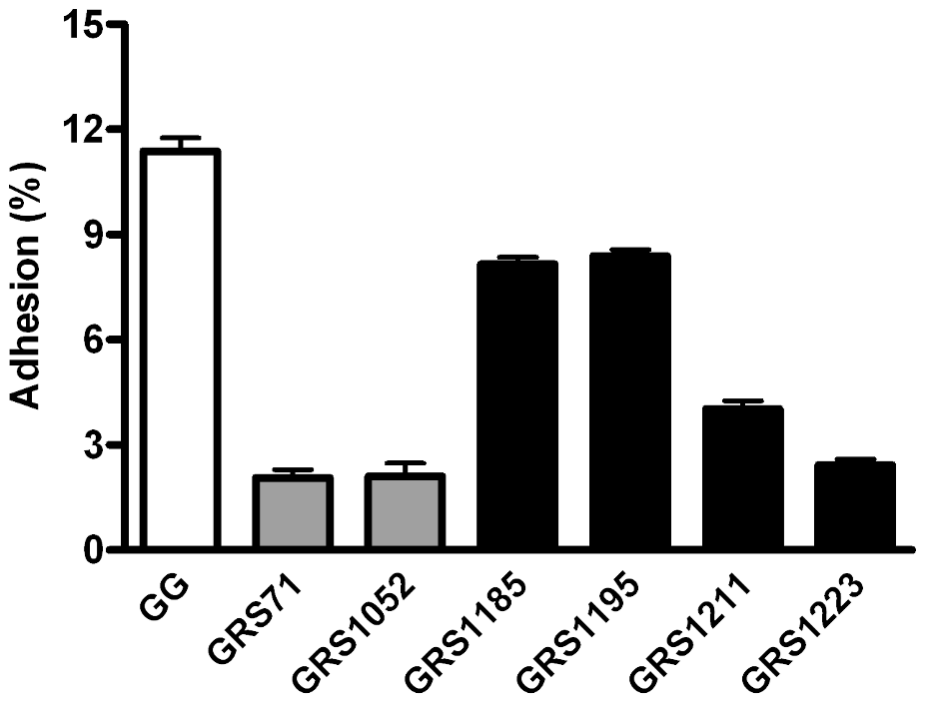
Binding of recombinant SpaCBA-piliated lactococcal cells to human intestinal mucus. The adhesion of metabolically labeled (^3^H) cells from normalized cultures of recombinant SpaCBA-piliated lactococcal constructs (GRS1185, GRS1195, GRS1211, and GRS1223), along with vectorless (GRS71) and empty vector (GRS1052) *L. lactis* NZ900 as negative controls and *L. rhamnosus* GG (GG) as a positive control, to human intestinal mucus was performed according to the method outlined in Materials and Methods. Measurements were done in quadruplicate and the experiment was performed three times. Error bars represent standard error of mean (SEM). Differences between data (GRS1185, GRS1195, or GRS1211) from pairwise comparisons against GRS71 data are regarded as extremely significant (*P*<0.001), with the exception of data from GRS1223, which was deemed not significant (*P*>0.05).

As indicated in Figure 2, nisin-induced GRS1185 cells producing WT SpaCBA pili could bind to human intestinal mucus at levels that, while not identical, were similar enough to that measured for *L. rhamnosus* GG cells (positive control). We reasoned that, despite the fact the number of cells had been normalized for the assay, the proportion of surface-localized pili per cell for these two piliated bacteria is variable and not matching, and thus obtaining mucus adherence at the exact same levels is likely a random or more so unachievable outcome. For instance, sometimes an inherent drawback of a peptide-inducible recombinant protein expression system is the stability of the inducing agent being used. Given that proteinaceous nisin is less stable at physiological pH (and here the pH of the growth medium used), it might be viewed somewhat less effective than, for example, a more robust and stable chemical inducer. Consequently, this could be one explanation for our electron microscopic observations of mixed populations of recombinant lactococci having a range of pili per cell (data not shown). Nonetheless, for the recombinant SpaCBA pilus itself, the level of binding to mucus is clearly higher than that obtained for the negative controls GRS71 and GRS1052. Thus, based on these results one can be reasonably assured that the recombinant SpaCBA pilus in *L. lactis* and the native form in *L. rhamnosus* GG share similar mucus adhesion functionalities.

Taking into account the adherent properties of SpaC and SpaB in *L. rhamnosus* GG as above described, we also assessed the functional changes imparted to the SpaCBA pilus in the three pilin deletion mutant constructs (GRS1195, GRS1211, and GRS1223) by measuring each of their abilities to bind mucus. For the nisin-induced GRS1211 (Δ*spaC*) and GRS1223 (Δ*spaB* and Δ*spaC*) constructs (Figure 2), each of which no longer encode for the SpaC pilin, both exhibited mucus adhesion at levels near the negative controls (see above), thus verifying that the functionality of SpaC is absent in the corresponding surface-expressed pili. However, for GRS1195 (Δ*spaB*) the deletion of SpaB did not seem to affect the mucus-binding capacity of this construct, whose own levels were nearly identical to that of GRS1185 and even though we reported previously that recombinant-produced SpaB protein could adhere to mucus [26]. However, we consider this finding fully consistent with our previous work [26], in which mucosal adhesion was not blocked when *L. rhamnosus* GG cells were pretreated with anti-SpaB serum, a result we had assumed was due largely to the less accessible location of SpaB at the pilus base. At any rate, the continued high level of mucus adherence observed with GRS1195, together with the results obtained with the GRS1211 and GRS1223 constructs, reaffirms the key role played by SpaC as the main mucus-binding determinant of the SpaCBA pilus.

### TLR2-mediated innate immune signaling by recombinant SpaCBA-piliated lactococcal constructs

The innate immune system relies on the ability of a relatively large collection of pattern recognition receptors (PRRs) to identify certain conserved bacterial ligands, known collectively as microbe-associated molecular patterns (MAMPs) [13], [37], [38]. Among the PRRs, TLR2 has specificity for a structurally diverse repertoire of MAMPs (i.e., peptidoglycan, lipoteichoic acid, and lipoprotein) in Gram-positive bacteria, which functionally is reliant on heterodimer formation with either TLR1 or TLR6 [13], [38]. Interestingly, a quite recent study has now demonstrated that the type 1 pilus from *Streptococcus pneumoniae* is a TLR2 agonist and can be considered an important determinant for TLR2-dependent induction by this particular Gram-positive pathogen [39]. Similarly, it has recently been inferred that the SpaCBA pilus in *L. rhamnosus* GG might be a determining factor that helps this bacterium trigger TLR2-activated NF-κB responses [28].

**Figure 3:**
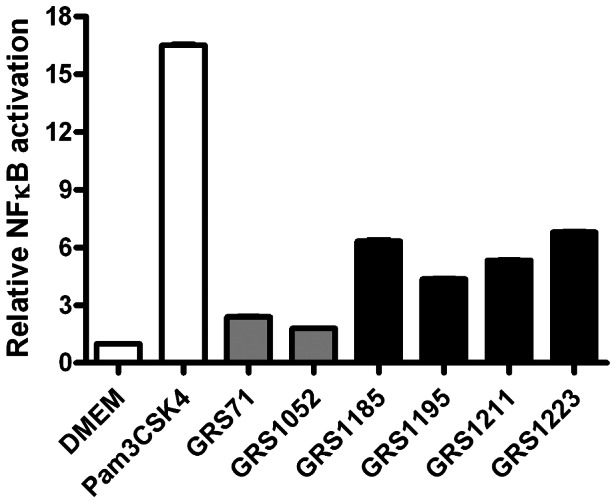
TLR2-mediated immune responses induced by recombinant SpaCBA-piliated lactococcal cells. Stimulation of TLR2-dependent NF-κB activation in the HEK-TLR2 cell line by normalized cultures of recombinant SpaCBA-piliated lactococcal cells (GRS1185, GRS1195, GRS1211, and GRS1223) was carried out according to the method described in Materials and Methods. GRS71 and GRS1052 lactococcal cells as well as DMEM cell culture media were included as negative controls. TLR2-agonist lipopeptide Pam3CSK4 (1 ng/ml) was used a positive control. Measurements were done in quadruplicate and the experiment was performed three times. Error bars are standard error of mean (SEM). Differences between data (GRS1185, GRS1195, GRS1211, or GRS1223) from pairwise comparisons against GRS71 data are considered extremely significant (*P*<0.001).

For this part of our study, we used the WT (GRS1185) and deletion mutant (GRS1195, GRS1211, and GRS1223) recombinant lactococcal constructs to assess whether the proteinaceous SpaCBA pilus can be recognized specifically by TLR2. Here, we initially examined the ability of nisin-induced GRS1185 to stimulate a stably co-transfected HEK293 cell line that contains the genes for human TLR2 and a NF-κB-regulated secreted alkaline phosphatase (SEAP) reporter system, the latter of which facilitates the monitoring of TLR2 signaling. Serving as a positive control, NF-κB activation was readily detected when HEK-TLR2 cells were treated with the TLR2-agonist lipopeptide Pam3CSK4 (1 ng/ml), while with the empty vector GRS1052 and vectorless GRS71 negative controls, NF-κB was activated at only low levels (Figure 3). However, NF-κB activation in HEK-TLR2 cells was considerably induced by the SpaCBA-piliated GRS1185 construct, thus indicating the specific stimulation of TLR2-dependent activity (Figure 3). Of note, it is worth mentioning that in similar experiments with “LPS-specific” HEK-TLR4 and “flagellin-specific” HEK-TLR5 cells, we observed that GRS1185-induced NF-κB activity was not detected (data not shown).

Despite the removal of the SpaB and SpaC pilins from the pili in GRS1195, GRS1211, and GRS1223 deletion mutants, all these constructs remained potent and still induced NF-κB activation in HEK-TLR2 cells at levels largely identical to that detected with WT-piliated GRS1185 (Figure 3). Such results would seem to suggest that TLR2 is specific for the recognition of the overall three-pilin macromolecular protein-fold architecture of polymerized subunits and less so, those individual minor pilin constituents located at the tip or base of the pilus structure. Then again, and somewhat supportive of this, it has recently been reported that while the tip-located adhesive pilin (RrgA) of the pneumococcal type 1 pilus is specifically responsible for TLR2-dependent activation, it was only optimal when this protein is in the structured form of macromolecular aggregates and not as monomeric protein [39].

**Figure 4:**
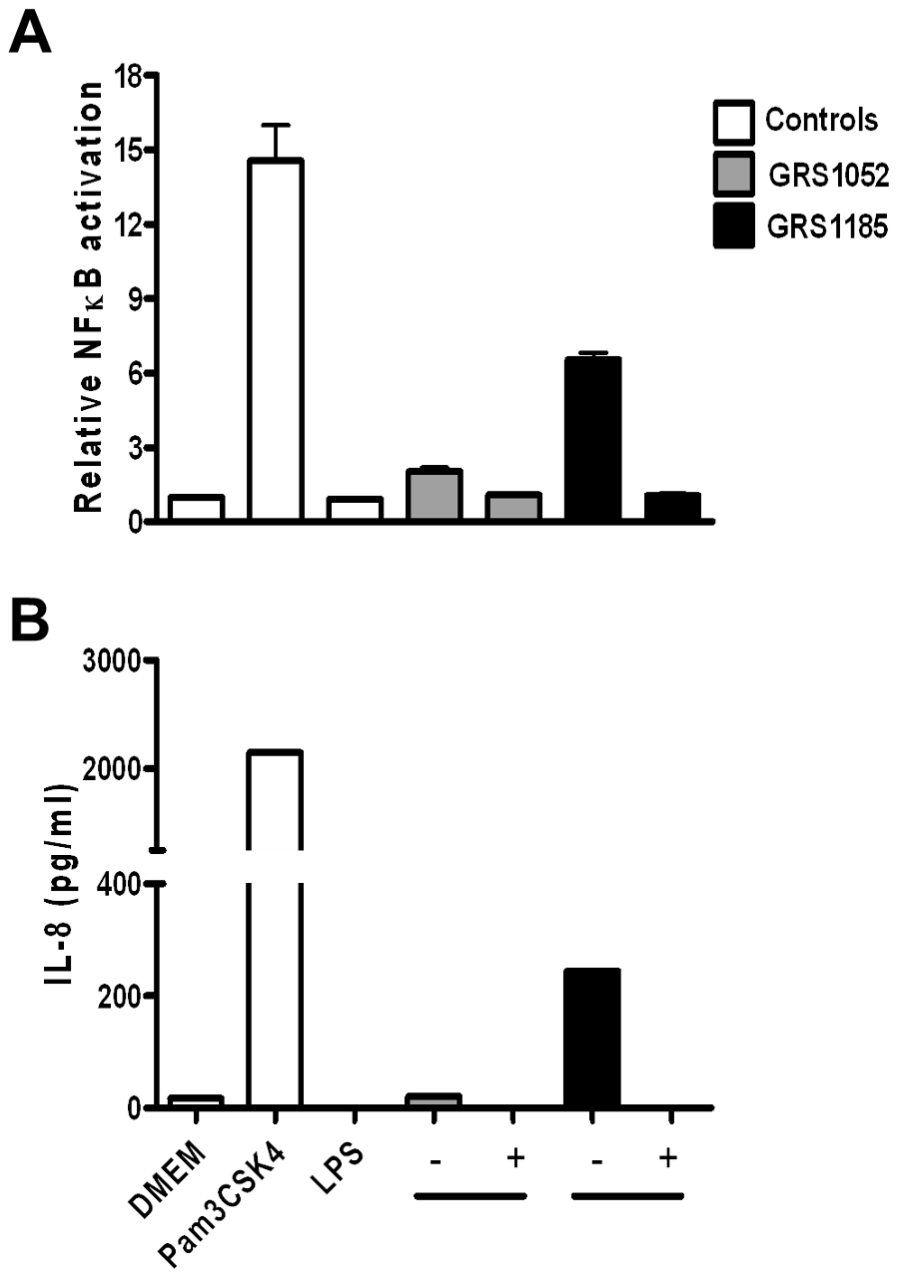
Effect of heat-treated WT SpaCBA-piliated lactococcal cells on TLR2-dependent immune activity. Stimulation of TLR2-dependent NF-κB activation (A) and IL-8 cytokine production (B) in the HEK-TLR2 cell line by either live (-) or heat-treated (100°C for 10 minutes) (+) WT SpaCBA-piliated lactococcal cells (GRS1185) was performed as described in Materials and Methods. Live and heat-treated GRS1052 lactococcal cells were also tested. DMEM cell culture media, Pam3CSK4 (1 ng/ml), and *E. coli* lipopolysaccharide (LPS; 1 ng/ml) were included as controls. Quadruplicate and duplicate measurements for NF-κB activation and IL-8 production, respectively, were performed. Error bars indicate standard error of mean (SEM). Differences between GRS1185 and GRS1052 data are deemed extremely significant (*P*<0.001).

To gain some indication whether the surface-associated factor in recombinant lactococci that augments TLR2-dependent activation is indeed proteinaceous in nature, we repeated the above experiment using heat treated nisin-induced GRS1185 bacteria (including those of GRS1052 as a control) (Figure 4). Besides monitoring NF-kB activation in the spent culture supernatant, we also checked for the production of IL-8, whose secretion occurs in various cell types, including epithelial cells, and which can be induced by the presence of microbial products. As detected with GRS1185, heat treatment sufficient to denature protein folding had noticeably reduced NF-κB activation in HEK-TLR2 cells (Figure 4A). Importantly, when live GRS1185 bacteria had been subjected to heat treatment, the normal induction in HEK-TLR2 cells of secreted IL-8 protein (an endogenous effector molecule of these cells) was then abolished (Figure 4B). Taken together, these results support the involvement of heat-labile proteins and might entail the participation of SpaCBA pili in triggering such TLR2 responses.

**Figure 5:**
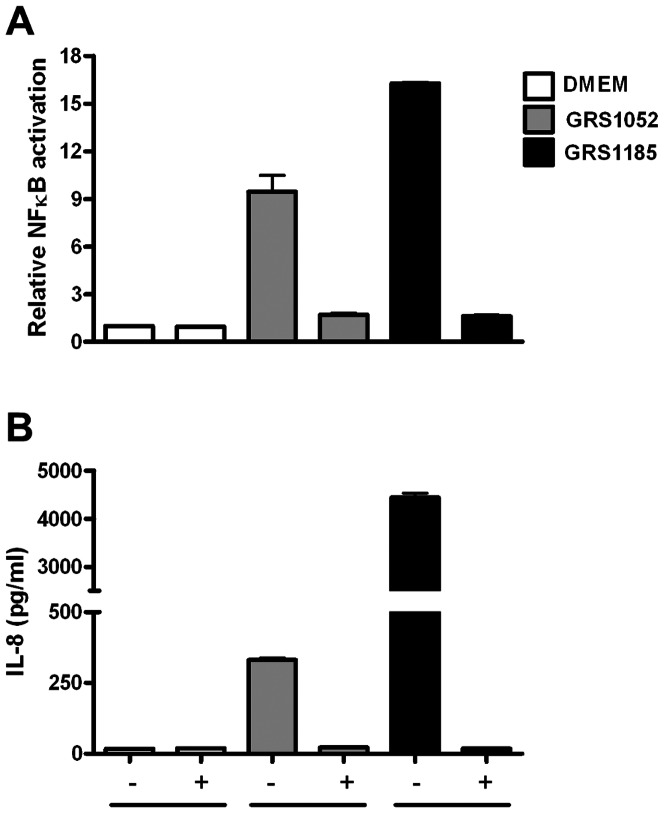
Influence of cell-to-cell interactions on TLR2-dependent immune responses induced by WT SpaCBA-piliated lactococcal cells. Stimulation of human TLR2-dependent NF-κB activation (A) and IL-8 cytokine production (B) in the HEK-TLR2 cell line by WT SpaCBA-piliated lactococcal cells (GRS1185; MOI 625), either non-partitioned (-) or partitioned (+) with Transwell membranes (0.4-µm pore size), was carried out as outlined in Materials and Methods. GRS1052 lactococcal cells (MOI 625) and DMEM cell culture media were included as negative controls. Measurements were conducted in quadruplicate and each experiment was carried out three times. Error bars reflect standard error of mean (SEM). Differences between GRS1185 and GRS1052 data are considered significant (*P*<0.05).

In an experiment to demonstrate whether these TLR2 actions rely on a surface-associated protein attached firmly to the cell wall as opposed to a protein product released from the recombinant lactococcal cells, we used Transwell cell culture membrane inserts (0.4-µm pore size) to partition the GRS1185 and HEK-TLR2 cells from each other, thus precluding any direct cell-to-cell contact. With the Transwell system (Figures 5A and 5B), both NF-κB activation and IL-8 production were only at the background levels of the DMEM control in those HEK-TLR2 cells separated from nisin-induced GRS1185 cells. Such results would seem to indicate the formation of local cell-to-cell interactions are needed for these TLR2-related immune responses, whose stimulation possibly involves a cell-wall anchored protein. Here, the SpaCBA pilus, via its adherent capacity, conceivably might help maintain a close association between the HEK-TLR2 and GRS1185 cells. In this regard, one might begin to ask whether such increased adhesion between “host immune” and “piliated target” cells can then lead to enhanced immunomodulatory activity in the context of the gut environment.

Speculatively, based on the ability of the SpaCBA-piliated lactococcal constructs to induce TLR2-dependent responses, it would seem reasonable to suggest that the surface-anchored SpaCBA pilus is most likely acting as a TLR2 agonist or activator. This would not only help corroborate the role of the SpaCBA pilus in the TLR2-dependent NF-κB activity of *L. rhamnosus* GG that had been inferred earlier [28], but also lend support to the putative TLR2-related functionality of SpaCBA pili in balancing the levels of LTA-induced IL-8 mRNA expression as had been demonstrated in a previous study [27]. Moreover, with this now the SpaCBA pilus can be included with cell wall LTAs in *L. rhamnosus* GG [22] as an immunomodulator with some element of discernible TLR2 agonist activity. However, most notably for this type of elongated surface-localized structure, our data supporting the possible new agonist function for the SpaCBA pilus in TLR2-regulated signaling would be in good agreement with what was reported recently (see above) for the type 1 pilus from the *S. pneumoniae* pathogen [39].

### Stimulation of DC cytokine production by recombinant SpaCBA-piliated lactococcal constructs

Antigen-presenting DCs are a heterogeneous population of immune cells that link innate and adaptive immune responses and, in effect, can help to control the homeostatic balancing of immune activation, tolerance, and suppression in the gut [37], [40]. DC activity can be mediated by the recognition of MAMPs in both commensal and pathogenic bacteria through a variety of host receptor systems, including the TLRs. Here, immature DCs undergo a maturation process that includes the up-regulated expression of cell-surface markers (e.g., CD83 and CD86), and where, in the end, mature DCs become very potent at cytokine production, which in itself is important for interactions among other immune cells [40]. Significantly, the role of DCs in the immune interactions of probiotic lactobacilli has been demonstrated [41], with the results from numerous studies having implicated some strains in also modulating DC surface expression and cytokine production (for review, see [9], [42], [43]).

**Figure 6:**
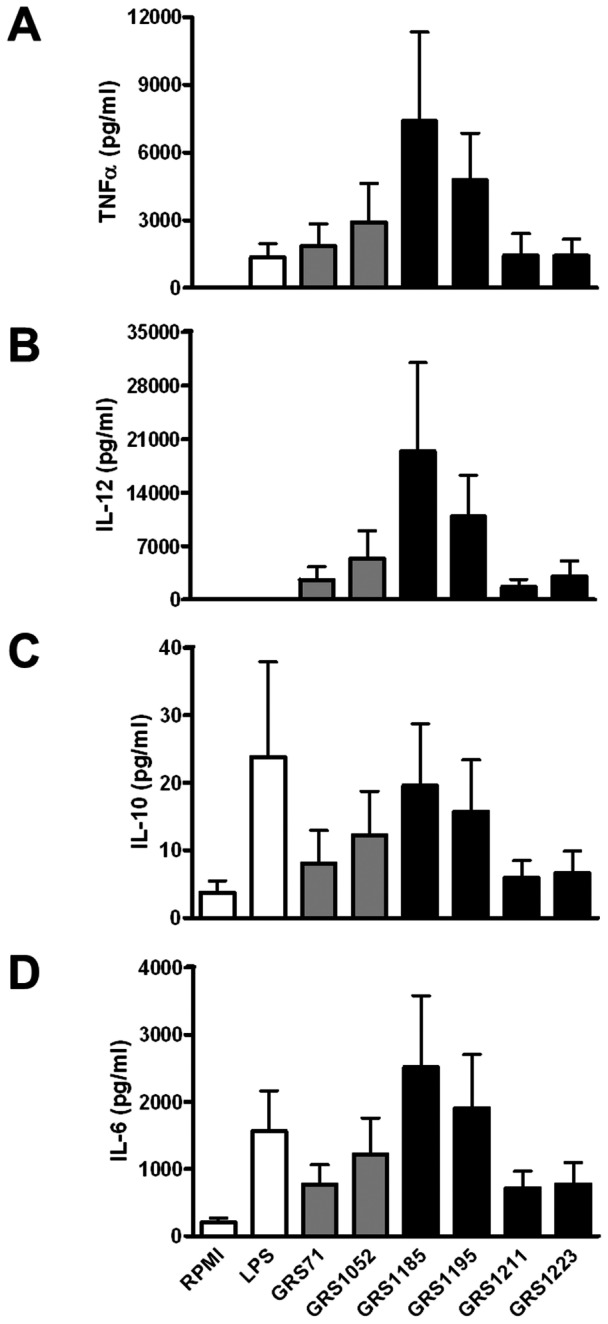
Induction of cytokine production in human moDCs by recombinant SpaCBA-piliated lactococcal cells. Stimulation of TNF-α, IL-12, IL-10, and IL-6 cytokine production (A, B, C, and D, respectively) in human moDCs by normalized cultures of recombinant SpaCBA-piliated lactococcal cells (GRS1185, GRS1195, GRS1211, and GRS1223) was carried out according to the method described in Materials and Methods. Cells from GRS71 and GRS1052 *L. lactis* were included as negative controls. RPMI cell culture media and *E. coli* lipopolysaccharide (LPS; 1 µg/ml) were also included as controls. Measurements were carried out in quadruplicate and each experiment was done three times with moDCs from four different donors, with the data used being the most representative of the experiments. Error bars indicate standard error of mean (SEM).

Accordingly, we decided to test the stimulatory effect of the recombinant SpaCBA-piliated lactococcal constructs on the production profile of various cytokines in human moDCs. In experiments performed with a predetermined optimal amount of lactococci (MOI 100), moDCs that were stimulated with SpaCBA-piliated GRS1185 cells had increased production levels for all four cytokines tested (i.e., TNF-α, IL-12, IL-10, and IL-6; Figures 6A-D, respectively). As to be anticipated, such production levels were not detected when moDCs were treated with the GRS71 and GRS1052 negative controls. Likewise, in comparison with the GRS1185 results, stimulation of moDCs by the three pilin deletion lactococcal constructs (GRS1195, GRS1211, and GRS1223) also give rise to reduced production of the four different cytokines. Noteworthy, however, the GRS1195 construct, which expressed SpaB-deleted pili, could stimulate cytokine production at about half the level seen with GRS1185-induced stimulation, but with the SpaC-deleted GRS1211 and SpaB- and SpaC-deleted GRS1223 constructs, cytokine induction in moDCs had only reached levels near what was observed for the negative controls. It would seem that because the removal of SpaC from the assembled pilus can cause a much more pronounced reduction in the levels of produced cytokines, this adherent pilin subunit might be an important determinant in SpaCBA pilus-induced cytokine responses by moDCs, and also what one might foresee is linked in part to its adhesive capacity.

Also in this part of the study, we decided to test whether the various SpaCBA-piliated lactococcal constructs would lead to differential DC maturation. Here, although the expression levels of three cell-surface marker proteins (CD83, CD86, and DC-SIGN) had changed when moDCs were stimulated with GRS1185 as opposed to GRS1052, on the whole these differences were only minimal (data not shown). Likewise, lactococcal cells with SpaCBA pili lacking SpaB or SpaC appeared to have no effect on the maturation profile (data not shown).

**Figure 7:**
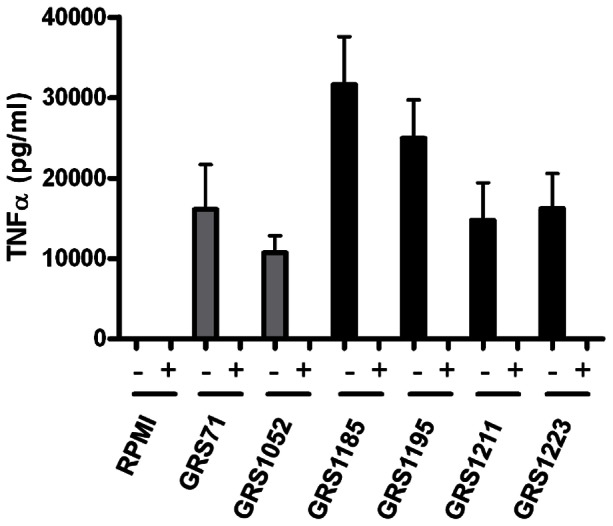
Effect of cell-to-cell interactions on TNF-α production in human moDCs induced by recombinant SpaCBA-piliated lactococcal cells. Stimulation of TNF-α production in human moDCs by normalized cultures of recombinant SpaCBA-piliated lactococcal cells (GRS1185, GRS1195, GRS1211, and GRS1223), either non-partitioned (-) or partitioned (+) with Transwell membranes (0.4-µm pore size), was done as outlined in Materials and Methods. GRS71 and GRS1052 lactococcal cells and RPMI cell culture media were included as negative controls. Measurements were performed in quadruplicate and the experiment was carried out with moDCs from four different donors. Error bars are standard error of mean (SEM).

Considering the commonly understood short half-life functionality of most cytokines and thus their need to be released locally to work effectively, it is generally requisite that a close proximity be maintained between host and target cells. With this in mind, one can again speculate that the adhesiveness property of SpaCBA pili could be related to the enhanced cytokine responses in the moDCs. This would be manifested by the improved cell-to-cell contact that is expected to occur when such immune cells are treated with SpaCBA-piliated lactococci rather than the nonpiliated form. In support of the above-stated idea, we performed an experiment to examine whether the induced DC secretion for one of the tested cytokines (i.e., TNF-α) would be affected when physical contact between moDCs and cells of the various recombinant lactococcal constructs is prevented. Once again, using the Transwell system to restrict direct cell-to-cell interactions, such that moDCs and WT or mutant SpaCBA-piliated lactococcal cells are placed in the lower and upper compartments, respectively, we found that we could no longer detect any TNF-α responses in this situation (Figure 7). We regard such results as indicative of a soluble factor not being involved in triggering cytokine production in these moDCs and instead that the juxtaposed positioning of host and target cells is essential. In such a context, this might mean a surface-localized and adhesive component like the SpaCBA pilus could help better position cells close enough together so that either pili themselves or in combination with some other surface molecules can perhaps act as the signaling stimuli for cytokine induction in moDCs.

In our experiments, we chose to examine a panel of pro- and anti-inflammatory cytokines that are among those used commonly for gauging what immunomodulating affect various commensal (and pathogenic) bacteria have on different DCs. Broadly interpreted, the pattern of up-regulated DC cytokine production we obtained with GRS1185 cells, and in correlation, the WT SpaCBA pilus, was generally balanced between pro-inflammatory (TNF-α, IL-6, and IL-12) and anti-inflammatory (IL-10) types. As an overall pattern of cytokine production, this has been basically seen in the findings of similarly performed studies involving commensals and herein our study suggests induced homeostatic basal-level responsiveness in moDCs when being challenged by the SpaCBA-piliated lactococci. However, surprisingly enough, while a previous study on the immunomodulatory action of *L. rhamnosus* GG had found this strain to be a relatively poor inducer of moDC cytokine responses [44], and to the same extent what was also observed in our study (data not shown), we showed that the recombinant lactococcal GRS1185 construct, or in effect the WT SpaCBA pilus, can stimulate a relatively marked increase in the production of the tested DC cytokines. Interestingly, *L. lactis* is itself reported to be a comparatively good inducer of cytokines in human- and murine-derived DCs [44], [45], but here in our study it would seem that the extra cell-surface localization of the SpaCBA pilus structure has an additive potentiating effect on lactococcal-induced changes in the cytokine production profile.

It is worthy of mention that it remains unclear to us the peculiarity of behavior attributed to *L. rhamnosus* GG in earlier work [44], as well as what we had also encountered (data not shown). Here, despite having such quasi “immuno-potent” SpaCBA pili of its own, *L. rhamnosus* GG can seemingly provoke only weak basal level moDC cytokine responses, those of which were much lower than what levels we observed being potentiated with WT SpaCBA-piliated lactococcal cells. However, be that as it may, the low cellular immunostimulating activity reported for *L. rhamnosus* GG generally falls within the realm of the borderline immune status of most gut commensal bacteria, and, as expected, is profoundly unlike the intense immunogenicity normally associated with many intestinal pathogens.

Then again, based on our previous electron microscopic observations [25], nothing seen visually (e.g., the EPS layer) on *L. rhamnosus* GG cells appears to hinder SpaCBA pili from being readily exposed exteriorly to the surrounding environment, thus fulfilling what can be considered one of the necessary physical preconditions for allowing a stimulatory effect on immune cells. For that reason, we are also inclined to consider that a certain degree of fluctuation or variation in protein production per cell can occur in different bacterial expression hosts, so that, for example, SpaCBA pili become more over-expressed in nisin-regulated recombinant lactococci than what is localized on the cell surface of *L. rhamnosus* GG by constitutive means. As such, this could be reflected in the magnitude of induced DC cytokine responses by these same piliated but different expression hosts. Still though, it is clear to us that the SpaCBA pilus, via its recombinant expression, has the unmistakable ability to potentiate further the level of immunogenic responsiveness by DCs already associated toward *L. lactis*.

## Conclusion

Since the rather recent discovery of pilus structures in *L. rhamnosus* GG, functional insights into the role of these so-called SpaCBA pili in the probiosis of this intestinal lactobacillar strain are only beginning to become more apparent. To further help in an understanding of their possible part played within the gut immune system, we engineered the functional recombinant expression of fully assembled WT and pilin-deleted SpaCBA pili in a *L. lactis* host strain. These piliated lactococcal constructs proved to be a useful tool for testing the stimulatory effects of SpaCBA pili on TLR2-dependent activities and on DC cytokine production. In so doing, this then has given us some indication of the possible immune function that surface piliation can perhaps offer *L. rhamnosus* GG. For instance, we suggest that the surface-anchored SpaCBA pilus, as expressed in recombinant form in lactococcal cells and in the context of its projected functioning in the gut, plausibly might be considered a type of MAMP-targeted modulator of innate immunity. Such a novel role would be consistent with the apparent functionality as a TLR2 agonist reported recently for the type 1 pilus from *S. pneumoniae* [39]. Thus, our results offer a degree of support for the importance of the SpaCBA pilus, particularly the SpaC adhesin pilin subunit, as a signaling entity that helps elicit innate immune activity.

Moreover, based on the findings of this current study and those of an earlier one [27], the SpaCBA pilus can be added to the growing number cell-surface features of *L. rhamnosus* GG that have an immunomodulating function (i.e., EPS [21], LTA [22], and the p75 and p40 proteins [17]-[20]). Accordingly, this would strengthen the evidence for the multifactorial nature of the host cell interplay of this particular strain. As a possible role *in vivo*, one can be speculative and envisage that through the basal stimulatory actions of SpaCBA pili on immune-related cells, for instance similar to what we observed with the moDC modulation of inflammatory cytokines, this type of cell-surface appendage might bring about a pre-sensitized immune condition that, in the end, can allow the host to better target and combat various gut-invading piliated pathogens. However, remaining unclear is in what manner and to what extent these surface-localized pili, implicit via a multifactorial contribution, would have as a moderating function in helping the gut immune system maintain the basal-level recognition and tolerance of *L. rhamnosus* GG as an overall less “immuno-intrusive” intestinal bacterium. Finally on this last point, and to conclude, it is expected that future studies on furthering such aspects should likely shed added light on the molecular immunogenicity of the SpaCBA pilus and by this then eventually provide a clearer picture whether this type of surface piliation does in fact contribute to the health-related functioning of *L. rhamnosus* GG in the human gut.

## Materials and Methods

### Bacteria, plasmids, growth media, and culture conditions

*Lactococcus lactis* NZ9000 (*pepN::nisRnisK*) [46], a derivative strain of *L. lactis* MG1363, and herein called GRS71, was grown overnight at 30°C with M17 medium (Difco) containing 0.5% glucose (GM17) on agar plates or statically in liquid broths, and was supplemented with 7.5 µg/ml chloramphenicol when needed for recombinant pilus production. *Lactobacillus rhamnosus* GG (ATCC 53103) was grown overnight at 37°C with de Man-Rogosa-Sharpe (MRS) medium (Difco) on agar plates or statically in liquid broths. Plasmid pKTH5080 (unpublished) was used for cloning in *L. lactis* and was derived from pNZ8032 [47], a lactococcal expression vector that contains regulatory genes (*nisR* and *nisK*) for nisin-inducible gene expression from the P*nisA* promoter, and which, for our cloning, was subsequently engineered to contain added sequences for the *Lactobacillus brevis* S-layer protein (SlpA) secretion signal and transcriptional terminator and for two additional restriction endonucleases (*Eco*RI and *Xho*I) in the multiple cloning region.

### DNA isolation and manipulations

The isolation of plasmid DNA from *L. lactis* using the QIAprep Spin Miniprep Kit (Qiagen) and genomic DNA from *L. rhamnosus* GG using the Wizard Genomic DNA Purification Kit (Promega) were both performed with slight modifications using the manufacturer-recommended protocols. Established DNA methods were used for molecular cloning, PCR amplification, restriction endonuclease digestion, DNA ligation, and other related techniques.

### Lactococcal cloning of expression constructs for SpaCBA pilus production

Construction of a lactococcal expression plasmid containing the *L. rhamnosus* GG four-gene *spaCBA* pilus operon, which includes the LGG_00444 (*spaC*), LGG_00443 (*spaB*), LGG_00442 (*spaA*), and LGG_00441 (*srtC1*) open reading frames (ORFs) [23], was carried out by overlap extension PCR (OE-PCR) [48] using oligonucleotide primers (Oligomer, Finland) listed in Table 1. With genomic DNA from *L. rhamnosus* GG as the template, an upstream OE-PCR fragment encoding about two-thirds of the *spaC* gene (including sequence for its own secretion signal peptide) was PCR amplified using forward primer 1986 and reverse primer 1988. A flanking downstream OE-PCR fragment encoding the remaining third of the *spaC* gene and all of the *spaB*, *spaA*, and *srtC1* genes was PCR amplified using forward primer 1985 and reverse primer 1989. Primers 1985 and 1988 were each designed to also introduce point mutations for the removal of an *Nco*I restriction site within the coding region of the *spaC* gene. The upstream and downstream OE-PCR fragments were then reconnected in-frame as the intact *spaCBA* pilus operon, which encodes for the SpaC, SpaB, and SpaA pilin subunits and the sortase-C1 enzyme, by a final PCR step using *Nco*I-containing forward primer 1984 and *Xho*I-containing reverse primer 1987. The final amplified PCR fragment, digested with *Nco*I and *Xho*I restriction endonucleases, was ligated into the pKTH5080 lactococcal nisin-inducible expression vector, which was subsequently electroporated into competent *L. lactis* NZ9000 cells as performed previously [49] and the transformants then selected for their antibiotic resistance on GM17 agar media containing 7.5 µg/ml chloramphenicol. PCR analysis and DNA sequencing confirmed transformant clones with plasmids containing the correct insert. A lactococcal clone with the right plasmid construct (designated pKTH5391) was chosen and called GRS1185.

**Table 1:** PCR oligonucleotide primers used for lactococcal cloning.

Primer	DNA Sequence (5' to 3')
1016	GTTCGGTACCATCAACAAAGCTCACCTA
1834	ACTTGAGATCTGACCTAGTCTTATAACTATACTGACAAT
1984	GTAAAATTTACGACCATGGCAGCTAAAGTG
1985	CAATGGCAATATCACGGCACAAAGGAC
1986	ATTGTCGCTTTACTGCGATGAAGCTTCTG
1987	GGATATTAAACAAATCTCGAGATCTTAATGATATTC
1988	GTCCTTTGTGCCGTGATATTGCCATTG
1989	AGACGGCTTCCACTATGGAAAGTCATC
1990	GACGGGTGGTGAAGCAACATGACTAAATCGCTCGGGTTGATAATCTTCGCGAC
1991	GTCGCGAAGATTATCAACCCGAGCGATTTAGTCATGTTGCTTCACCACCCGTC
2109	CTGGGCATTTGTTCGCGGTCTTATTGATTGCACTGGGATTGATCAGCGCAGCGTTC
2110	GAACGCTGCGCTGATCAATCCCAGTGCAATCAATAAGACCGCGAACAAATGCCCAG

Sequences for *Nco*I (1984) and *Xho*I (1987) restriction endonucleases used to facilitate cloning are underlined

A similar OE-PCR approach using genomic DNA was also undertaken to generate lactococcal clones expressing SpaB or SpaC pilin-deleted SpaCBA pili. A plasmid construct (pKTH5497) that lacks the *spaB* gene in the *spaCBA* pilus operon was obtained by connecting an upstream fragment encoding *spaC* (PCR amplified with forward primer 1986 and reverse primer 1981) to a downstream fragment encoding a 16-residue C-terminal portion of *spaB* and the *spaA* and *srtC1* genes (PCR amplified with forward primer 1990 and reverse primer 1989) by PCR amplification with primers 1984 and 1987. For generating the *spaC*-deleted plasmid construct (pKTH5413), pairwise 1986 (forward) and 2110 (reverse) primers and pairwise 2109 (forward) and 1989 (reverse) primers were used for obtaining their respective upstream and downstream PCR fragments, which then were PCR amplified with primers 1984 and 1987. The *spaB*-*spaC* double-deletion plasmid construct (pKTH5440) was generated using pKTH5413 plasmid DNA as the template. Here, an upstream PCR fragment, which was PCR amplified with forward primer 1834 and reverse primer 1991, and a downstream PCR fragment, which was PCR amplified with forward primer 1990 and reverse primer 1016, were reassembled by PCR with primers 1834 and 1016. Final amplified PCR fragments representing the three different pilin deletion constructs were first digested with *Nco*I and *Xho*I and then each was ligated into pKTH5080, after which all were transformed into *L. lactis* NZ9000 and selected as above described. Following their confirmation by PCR screening and DNA sequencing, the resulting lactococcal mutant clones were called GRS1195 (Δ*spaB*), GRS1211 (Δ*spaC*), and GRS1223 (Δ*spaB* and Δ*spaC*). *L. lactis* GRS1052 (unpublished), which carried the empty vector pKTH5080, was used as a control.

### Lactococcal production of recombinant SpaCBA pili

Starter cultures of recombinant *L. lactis* constructs (GRS1185, GRS1195, GRS1211, GRS1223, and GRS1052) were normally grown statically overnight at 30°C in GM17 liquid broths that included 7.5 µg/ml chloramphenicol, and after being diluted 1:200 in the fresh tubes of the same growth medium, were grown at 30°C to an optical density at 600 nm (OD600) of about 0.4. At this point, pilus protein production was induced by adding nisin (∼0.5 to 5 ng/ml) to the culture, which was then allowed to continue growing overnight. Cells were recovered by low speed centrifugation (∼5,000 × *g*), after which cell pellets were resuspended in a buffer suitable for their intended use.

### Immunoblotting and immuno-electron microscopy

Nisin-induced cultures of *L. lactis* constructs were cell fractionated according to a protocol described earlier for *L. rhamnosus* GG [25]. Isolated cell wall-bound proteins were separated by SDS-PAGE and pili were detected by immunoblotting with antiserum raised against recombinant SpaCBA pilin proteins [26] as described previously [25]. Immunogold labeling of SpaCBA-piliated cells (recombinant lactococcal constructs and *L. rhamnosus* GG) and transmission electron microscopic analyses were performed essentially as described previously [25].

### Mucus adhesion assays

Human intestinal mucus served as the substrate for adhesion assays (whose use was established previously [50]) and was obtained surgically as resected tissue from patients with operable colorectal cancer. As described previously [51], the mucus gel layer was recovered from sections of noncancerous tissue containing intact mucosa. The adhesion of nisin-induced recombinant lactococcal cells to intestinal mucus was carried out according to a method described earlier [23], [26], in which for the assay used the cells are metabolically radiolabeled with tritiated thymidine and the cell numbers are normalized to an OD600 of 0.25. A detailed description of the assay conditions and protocol has been provided elsewhere [23], [26].

### Ethics Statement

The Hospital District of Southwest Finland ethics committee authorized and approved the use of human tissue specimens for mucus isolation and those patients involved had been informed beforehand and given their written consent.

### Stimulation of human HEK-TLR2 cells

The HEK293 cell line harboring human TLR2 and the secreted embryonic alkaline phosphate (SEAP) reporter gene fused to NFκB was purchased commercially as HEK-Blue™-hTLR2 from InvivoGen (USA). Cells were maintained and stimulated using the culture media conditions specified by the manufacturer. For this, Dulbecco's modified Eagle’s media (DMEM) containing 4.5 g/l glucose was supplemented with 10% (v/v) heat-inactivated fetal calf serum (FCS), 50 U/ml penicillin, 50 µg/ml streptomycin, 2 mM L-glutamine, 100 µg/ml Normocin™, and, when required, HEK-Blue™ selection antibiotics. HEK-TLR2 cells were seeded at cell densities of 5.0×10^4^ and 2.0×10^5^ in 96- and 24-well plates, respectively, using culture media with no selection antibiotics. Cells from overnight cultures were stimulated with bacteria at different multiplicity of infection (MOI) values (0.1 to 1000). NF-kB activation was observed to occur in a dose-dependent manner, with the amount of bacteria triggering the best response (MOI 100) then being chosen for use in the different experiments, unless otherwise indicated. TLR2-stimulated activation of SEAP production in culture supernatants was itself determined spectrophotometrically at 620 nm using QUANTI-Blue™ detection medium according to the instructions recommended by the manufacturer. Briefly, 20 µl of the stimulated cell culture media (done in quadruplicate per different sample) were added to the 96-well plate containing 180 µl of QUANTI-Blue reagent, which was then incubated at 37°C. Color development was monitored and the spectrophotometric measurements taken at 15-, 60-, 120-, and 180-minute time points. Production of IL-8 in HEK-TLR2 cells was measured using the BD OptEIA™ ELISA kit according to the protocol recommended by the manufacturer (BD Biosciences).

### Isolation and generation of human moDCs

Purified primary monocytes were recovered from fresh leukocyte-rich buffy coats, obtained from the donated blood of healthy humans (Finnish Red Cross Blood Transfusion Service), according to a method described earlier [52], [53]. For this, removal of red blood cells and the subsequent isolation of peripheral blood mononuclear cells were carried out by using density gradient centrifugation on Ficoll-Paque (GE Healthcare) with Leucosep separation tubes (Greiner Bio-One, Germany), which was then followed by Percoll™ (GE Healthcare) gradient centrifugation. Any remaining T- and B-cell lymphocytes were separated away by using anti-CD3 and anti-CD9 magnetic beads (Dynal Invitrogen, Norway). Isolation of monocytes was based on their plastic surface-binding properties and carried out by allowing them to adhere to 6- or 24-well cell culture plates for one hour at 37°C with 5% CO_2_, after which any nonadhering cells were aspirated away and those cells still bound were rinsed twice with PBS. Adherent monocytes were maintained for six days and allowed to differentiate into dendritic cells in Roswell Park Memorial Institute (RPMI) 1640 media (Sigma) containing 10% (v/v) FCS and supplemented with 0.6 µg/ml penicillin, 60 µg/ml streptomycin, 2 mM L-glutamine, 20 mM HEPES, 10 ng/ml granulocyte macrophage-colony stimulating factor (GM-CSF), and 20 ng/ml IL-4 (GM-CSF and IL-4 were from Gibco, Sweden). Cells were replenished with fresh media (1 ml/well) every two days. Cells had displayed typical DC morphology and were found to be positive for CD80 and CD86, but negative for CD14 and CD83 cell-surface marker proteins (data not shown). Experiments were performed with four different donors in RPMI 1640 media containing 10% FCS, antibiotics, L-glutamine, and HEPES. Stimulated cells and corresponding cell culture supernatants from donor samples were collected and analyzed individually.

### Stimulation of human moDCs

Cell suspensions of *L. lactis* and *L. rhamnosus* GG were normalized (OD600) in RPMI 1640 media containing 10% FCS, antibiotics, L-glutamine, and HEPES. Human moDCs were treated with various amounts of bacteria (e.g., MOI 1, 10, and 100) and allowed to incubate at 37°C with 5% CO_2_ for about 24 hours. Cell culture supernatants were then collected and measured for cytokines (TNF-α, IL-6, IL-10, and IL-12) using the Bio-Plex Pro cytokine assay in the Bio-Plex™-200 system according to manufacturer recommended instructions (Bio-Rad, UK).

### Transwell assay analysis

Transwell (Corning, USA) cell culture membrane inserts (0.4-µm pore size) were used to partition bacterial and human cells from each other so as to prevent any direct physical interactions. For this, the HEK-TLR2 cell line was seeded (2.0×10^5^) at the bottom of 24-well plates and then allowed to grow overnight at 37°C in a 5% CO_2_ incubator. On the following day, overnight-grown bacterial cells were rinsed twice with PBS and the cell numbers were then adjusted to an OD600 of 0.5 (*L. lactis*) or 1.0 (*L. rhamnosus* GG), with each being considered equivalent to 1.0×10^9^ cells/ml. Bacteria were added as a 100-µl volume directly to wells containing the HEK-TLR2 cells or first to the transwell insert compartment that was then submerged in wells with HEK-TLR2 cells. After ∼24-hour incubation at 37°C with 5% CO_2_, each of the supernatants was collected for QUANTI-Blue and IL-8 ELISA analyses. Individual sample measurements were done in quadruplicate for the QUANTI-Blue determinations. For transwell experiments with human moDCs, two 24-well plates, one with transwell inserts (Becton Dickinson & Company, USA) and another without, had been used, each of which contained an initial monocyte density of ∼1.25×10^6^ cells. DCs were stimulated similarly with bacterial cells (see above) and cell culture supernatants then collected for ELISA measurements.

### Statistical analysis

All statistical analyses of data were performed using GraphPad Prism (version 4.0). To support the statistical relevance of data from experiments measuring mucus adhesion (Figure 2) and induced TLR2-dependent activity (Figures 3-5), pairwise comparisons were made using the unpaired Student’s *t* test. Calculated *P* values were deemed significant (0.05 or less) or extremely significant (0.001 or less). Donor-specific variation in moDCs had prevented the reliable estimation of similar statistical measurements for the cytokine production data in Figures 6 and 7.
